# Effect of temperature cycles on the sleep-like state in *Hydra vulgaris*

**DOI:** 10.1186/s40851-025-00248-1

**Published:** 2025-01-28

**Authors:** Aya Sato, Manabu Sekiguchi, Koga Nakada, Taishi Yoshii, Taichi Q. Itoh

**Affiliations:** 1 Faculty of Arts and Science, Kyushu University, Fukuoka, 819-0395 Japan; 2 Graduate School of Systems Life Sciences, Kyushu University, Fukuoka, 819-0395 Japan; 3 Graduate School of Natural Science and Technology, Okayama University, Okayama, 700-8530 Japan; 4 Graduate School of Environmental, Life, Natural Science and Technology, Okayama University, Okayama, 700-8530 Japan

**Keywords:** *Hydra*, Sleep, Temperature, Environmental cues

## Abstract

**Background:**

Sleep is a conserved physiological phenomenon across species. It is mainly controlled by two processes: a circadian clock that regulates the timing of sleep and a homeostat that regulates the sleep drive. Even cnidarians, such as *Hydra* and jellyfish, which lack a brain, display sleep-like states. However, the manner in which environmental cues affect sleep-like states in these organisms remains unknown. In the present study, we investigated the effects of light and temperature cycles on the sleep-like state in *Hydra vulgaris*.

**Results:**

Our findings indicate that *Hydra* responds to temperature cycles with a difference of up to 5° C, resulting in decreased sleep duration under light conditions and increased sleep duration in dark conditions. Furthermore, our results reveal that *Hydra* prioritizes temperature changes over light as an environmental cue. Additionally, our body resection experiments show tissue-specific responsiveness in the generation ofthe sleep-like state under different environmental cues. Specifically, the upper body can generate the sleep-like state in response to a single environmental cue. In contrast, the lower body did not respond to 12-h light–dark cycles at a constant temperature.

**Conclusions:**

These findings indicate that both light and temperature influence the regulation of the sleep-like state in *Hydra*. Moreover, these observations highlight the existence of distinct regulatory mechanisms that govern patterns of the sleep-like state in brainless organisms, suggesting the potential involvement of specific regions for responsiveness of environmental cues for regulation of the sleep-like state.

**Supplementary Information:**

The online version contains supplementary material available at 10.1186/s40851-025-00248-1.

## Background

Sleep is a fundamental aspect of animal life and is tightly regulated by interactions between external cues and internal mechanisms. Sleep states exhibit rhythmic characteristics, such as behavioral quiescence and altered responsiveness to external stimuli [1, 2]. The two-process model, which is a widely accepted hypothesis, proposes that sleep regulation is the result of interaction between sleep pressure and the circadian clock. Sleep pressure increases during wakefulness and decreases during sleep. Sleep is induced when the pressure reaches a high threshold. As sleep continues, the pressure decreases to a lower threshold, prompting wakefulness. The circadian clock helps estimate these cycles within a 24-h period, ensuring that sleep occurs at an appropriate time [3]. However, despite valuable insights provided by the hypothetical two-process model, several key questions remaining unanswered, particularly in the context of a sleep-like state in organisms that lack a circadian clock.

The small freshwater cnidarian *Hydra* is commonly used as model organism in research areas, such as regeneration, stem cell differentiation, aging, and symbiosis [4]. Many of its fundamental body plans and genetic mechanisms are conserved across other animal groups. *Hydra* serves as an appropriate model for understanding the evolutionary origins of mechanisms underlying environmental responses and behavior. one recent study reported a sleep-like state in *Hydra*, which lacks both a brain and a circadian clock. Furthermore, it indicated that the molecular mechanism of this sleep-like state is regulated by neurotransmitters, similar to that in mammals and other organisms [5]. However, despite these advances, our understanding of the environmental factors that influence the sleep-like state in *Hydra* remains limited.

Temperature is a critical synchronizing factor in a variety of organisms, including mammals, chickens, zebrafish, and fruit flies [6–9]. Notably, even minor temperature differences can synchronize behavioral patterns in flies [10]. In addition to their impact on the circadian clock, temperature fluctuations have also been shown to influence the structure of sleep [11, 12], suggesting the importance of temperature as a sleep regulator, comparable to light.

Light- and temperature-sensing mechanisms in *Hydra* have been identified to some extent, providing insights into their responsiveness to environmental cues. For instance, *Hydra* possesses 45 conserved rhodopsins, which suggests potential roles in photosensing [13]. Molecular phylogenetic analyses further suggested their contributions to light perception. Regarding temperature sensitivity, at least 34 transient receptor potential channels have been reported in *Hydra*, indicating their involvement in temperature detection [14]. Changes in neural activity in response to temperature fluctuations have also been documented [15], indicating an intricate interplay between environmental cues and neural processing in *Hydra*.

Given the significance of temperature in regulating sleep behaviors in other organisms and the light- and temperature-sensing mechanisms identified in *Hydra*, it is crucial to investigate the influence of temperature cycles on the sleep-like state in *Hydra*. In the present study, we sought to explore the impact of temperature fluctuations on the sleep-like state in *Hydra* and elucidate the potential involvement of specific regions participating in receiving environmental cues for generating sleep-like state through resection experiments under different environmental conditions.

## Materials & methods

### Animals

*Hydra vulgaris* (*strain 105*) individuals without buds were used in all experiments. The *Hydra* were maintained in a hydra culture solution (HCS; 1 mM NaCl, 1 mM CaCl_2_, 0.1 mM KCl, 0.1 mM MgSO_4_, 1 mM tris-(hydroxymethyl)-amino-methane; pH 7.4, adjusted with HCl) at 20 °C under a 12-h light–dark cycles (LD 12:12 cycles). Light intensity during the light phase was maintained at approximately 450 lx. The *Hydra* were fed newly hatched *Artemia* nauplii twice per week.

### Behavioral tracking and data analysis

*Hydra* were subjected to a 36-h starvation period prior to behavioral recording. Each *Hydra* was placed in a square silicone container (16 mm × 16 mm × 5 mm) filled with 1 mL of HCS. The *Hydra* were placed in an incubator at zeitgeber time 10 (ZT10: ZT0 and ZT12 represent the time when the light was turned on and off, respectively), and video recording was initiated simultaneously. To allow acclimation to the environment, the sleep analysis was conducted starting from ZT0 the following day. To permit the continuous recording of *Hydra* behavior under both light and dark conditions, *Hydra* were illuminated with infrared light at a wavelength of 850 nm (Session Digital, Tokyo, Japan) and visualized using an E3 CMOS (complementary metal oxide semiconductor) camera (Visualix, Hyogo, Japan) through an infrared high-pass filter (FUJIFILM, Tokyo, Japan). For the LD cycles, a white-emitting electrode LEDSC-190-W (MiSUMi, Tokyo, Japan) was utilized at a light intensity of 680 lx. Temperature cycles (TC) were regulated using a program in an CN-40A incubator (Mitsubishi Electric, Tokyo, Japan). Frames were captured every 5 s at a resolution of 1920 × 1200 pixels and saved with an 8-bit grayscale resolution. A custom ImageJ macro was employed for frame subtraction analysis to quantify *Hydra* behavior, as previously described [16]. Sleep parameters were quantified in accordance with previously described methodology [5]. For quantification of total activity of *Hydra*, average pixel changes during the two experimental days were calculated.

### *Hydra* resections

*Hydra* resections were conducted approximately one to two hours prior to the commencement of behavioral tracking. *Hydra* were placed on glass slides during the resection procedure. The *Hydra* were permitted to relax for a few minutes, after which the resections were performed using a scalpel. Initially, the central region of the *Hydra* was severed, leaving only the upper and lower body intact. Untreated *Hydra* served as the control. Including the acclimation period immediately preceding the behavioral recording, the *Hydra* has sufficient time to close their wounds, yet they were unable to restore the functionality of the lost body parts or nerve cells [17].

### Measurement of arousal threshold

Each *Hydra* was placed into a rectangular silicone container (16 mm × 16 mm × 5 mm) filled with 1 mL of HCS at either 10 or 20 °C. During the dark phase, five light stimuli of 1400 lx were applied at 2-h intervals, each lasting 30 s. *Hydra* behavior was recorded before and after light stimulation, considering *Hydra* that remained stationary for at least 20 min before light stimulation were considered to be sleeping. The proportion of sleeping *Hydra* that awakened within 10 min of each light stimulus was calculated.

### Statistical analysis

Statistical analyses were conducted using R software (version 4.3.3). Normality was assessed for the two sample groups using the Shapiro–Wilk normality test conducted at a significance level of 0.05. In cases where normality was rejected, the Mann–Whitney U test was employed. To compare the paired sample groups, the Wilcoxon signed-rank test was utilized due to violations of the assumption of normality of the dataset. For multiple comparison tests, normality was assessed using the Shapiro–Wilk test at a significance level of 0.05. When normality was rejected, the Kruskal–Wallis test was conducted, followed by the Mann–Whitney U test with Bonferroni correction or Dunn-Bonferroni post-hoc test was conducted. Fisher's exact test was used to compare the proportion of *Hydra* awakening after stimulation.

## Results

### *Hydra* show day-night variation in the sleep-like state under cold temperature

**Fig. 1:**
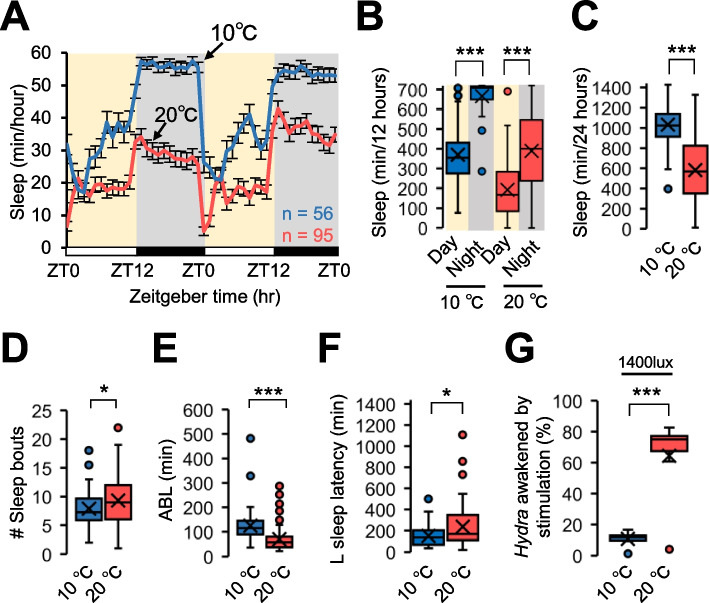
The sleep-like state under cold temperature. A Daily sleep profiles under 10 °C (Blue line) and 20 °C (Red line) represent mean ± SEM (*n* = 56–95). Box plots represent **B** sleep amount in day and night (n = 56–95), **C** Total sleep amount (*n* = 56–95), **D** Total number of sleep bouts (*n* = 56–95), **E** Averaged sleep bout length (ABL) (*n* = 56–95), and **F** L latency (*n* = 56–95). **G** Percentage of *Hydra* awakened by stimulation under 10 °C and 20 °C conditions. Stimulation was 1400 lx. Represent mean ± SEM (*n* = 5). *P < 0.05, ***P < 0.001, by Kruskal–Wallis test followed by Mann–Whitney U test with Bonferroni correction [B-D], Mann–Whitney U test [E], and Fisher’s exact test [G]. LD12:12, 12-h/12-h light–dark

To investigate the effect of temperature variation on the sleep-like state of *Hydra*, we conducted behavioral analyses under LD12:12 conditions at both 10 and 20 °C. Irrespective of temperature, day-night variation in the sleep-like state was observed, with an increase in sleep duration in darkness compared to the light condition (Fig. 1A, B). Furthermore, a significant increase in total sleep was observed at 10 °C in comparison to that at 20 °C (Fig. 1C). Furthermore, other sleep parameters were examined. The number of sleep bouts, defined as the number of sleep episodes, was found to be lower at 10 °C, compared to 20 °C (Fig. 1D). Moreover, the average sleep bout length (ABL), representing the average duration of individual sleep bouts, was found to be significantly longer in *Hydra* at 10 °C than at 20 °C (Fig. 1E). Additionally, the latency to sleep onset following light transitions, referred to as L sleep latency, demonstrated a significant decrease in 10 °C, compared to 20 °C (Fig. 1F). Furthermore, we examined the arousal thresholds of *Hydra* at 10 °C and 20 °C were examined by applying light stimuli of 1400 lx at night. The proportion of *Hydra* that were awakened within 10 min of light stimulation was significantly lower at 10 °C than at 20 °C (Fig. 1G). Consequently, the diurnal variations in the sleep-like state exhibited by *Hydra* under cool temperatures were observed to include longer sleep-like state, and less sensitivity to stimuli.

### The sleep-like state in *Hydra* is influenced by temperature cycles

**Fig. 2:**
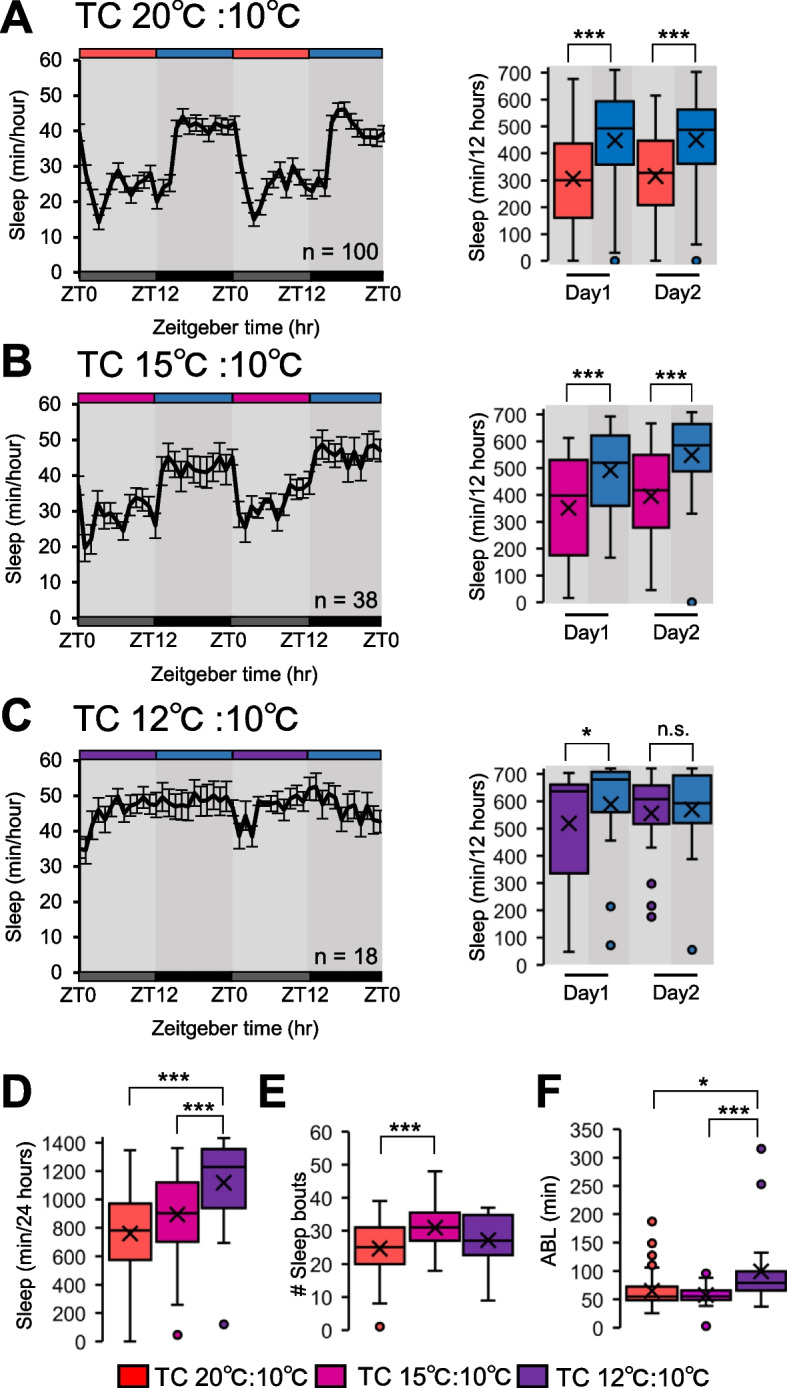
The sleep-like state response to temperature cycles. **A**-**C** Daily sleep profiles under DD with temperature cycles (A:20 °C/10 °C (*n* = 100), **B**:15 °C /10 °C (*n* = 38), **C**: 12 °C/10 °C (*n* = 18) represent mean ± SEM. Box plots represent sleep amount. **D**-**F** Box plots represent (**D**) Total sleep (*n* = 18–100, (**E**) number of sleep bouts (*n* = 18–99), (**F**) ABL (*n* = 18–99). n.s., not significant. *P < 0.05, ***P < 0.001 by Wilcoxon signed-rank sum test [A-C]. *P < 0.05, **P < 0.01 ***P < 0.001 by Kruskal–Wallis test followed by Mann–Whitney U test with Bonferroni correction [D-F] DD, constant darkness; TC, temperature cycles

We next investigated how *Hydra*, which lacks a conventional circadian clock, responds to TC cycles. To investigate this, a behavioral analysis was conducted under constant darkness in a 20 °C:10 °C TC cycles environment. The results demonstrated that the net sleep duration was significantly lower during the warmer period and significantly higher during the cooler period of the TC cycles (Fig. 2A). Having established that *Hydra* respond to TC cycles, subsequent behavioral analyses were conducted under 15 °C:10 °C and 12 °C:10 °C TC cycles to determine the extent of temperature differences necessary to alter the sleep-like state in response to the TC cycles. In the 15 °C:10 °C TC cycles environment, the sleep amount was significantly lower during the warmer period and significantly higher during the cooler period (Fig. 2B). In contrast, in the 12 °C:10 °C TC cycles environment, the difference in the sleep duration between the warmer and cooler periods was considerably smaller, with no significant difference observed on the second day (Fig. 2C). We further conducted a detailed analysis of the sleep parameters. The total sleep increased as the temperature difference decreased (Fig. 2D). Although the significant difference was observed only in the number of sleep bouts between 20 °C:10 °C and 15 °C:10 °C TC cycles, ABL was significantly higher in the 12 °C:10 °C TC cycles environments compared to the other conditions (Fig. 2E, F). These findings indicate that *Hydra* are capable of perceiving different temperature ranges, but a difference of at least 5 °C is necessary to respond to TC cycles. A 2 °C difference does not consistently produce a significant difference in the total sleep between 12 °C and 10 °C in TC cycles. Furthermore, our comparison of waking activity levels during the 20 °C:10 °C TC cycles revealed a significant reduction in activity at 10 °C compared to 20 °C. This suggests that the sleep-like state at lower temperatures may be influenced by reduced metabolic activity (Supplementary Fig. 1). Nonetheless, the presence of this sleep-like state has been confirmed through behavioral assessments, even at low temperatures.

### *Hydra* prioritize temperature over light as an environmental cue

**Fig. 3:**
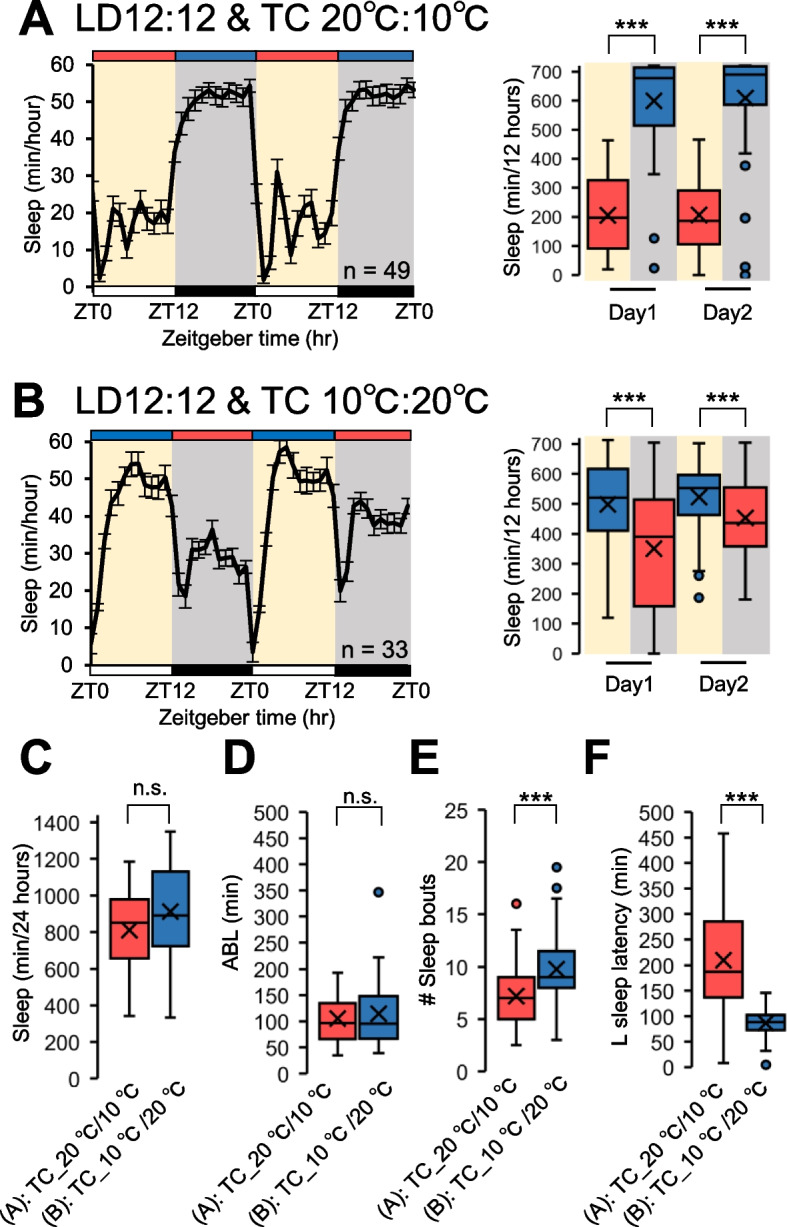
Aligned environmental cycles enhance quality of the sleep-like state. **A** Daily sleep profiles under LD12:12 with temperature cycles (20 °C/10 °C). Represent mean ± SEM (*n* = 49). Box plots represent sleep amount. **B** Daily sleep profiles under LD12:12 with temperature cycles (10 °C/20 °C) represent mean ± SEM (*n* = 33). Box plots represent sleep amount. **C** Total sleep (*n* = 33–49). **D** ABL (*n* = 33–49), **E** number of sleep bouts (*n* = 33–49), **F** L sleep latency (*n* = 33–49). n.s., not significant. ***P < 0.001 by Wilcoxon signed-rank sum test [A-C]. ***P < 0.001 by Mann–Whitney U test [D-F]. LD, 12-h/12-h light–dark; TC, temperature cycles

*Hydra* exhibited a sleep-like state in response to both the light and temperature cycles. Therefore, we conducted an investigation to determine the effect of simultaneous light and temperature cycles on the sleep-like state. For this purpose, behavioral analyses were conducted in environments where light and temperature were aligned, with high temperatures during the light phase and low temperatures during the dark phase, as well as in environments with misaligned cycles, featuring low temperatures during the light phase and high temperatures during the dark phase. In both environments, the sleep amount during the low-temperature phase was significantly higher than that during the high-temperature phase (Fig. 3A, B). This suggests that the *Hydra* prioritizes temperature over light. A comparison of the sleep parameters at both environments revealed no significant difference in the total sleep and ABL. However, the number of sleep bouts was significantly lower in the aligned environment (Fig. 3C, D, E). Furthermore, the L sleep latency was significantly shorter in *Hydra* exposed to environments with misaligned environmental cycles than in those exposed to aligned cycles (Fig. 3F). Additionally, when *Hydra* were shifted from LD and TC (LDTC) cycles to constant conditions, the patterns of the sleep-like state disappeared (Supplementary Fig. 2). This result indicates that the observed rhythmic changes are closely tied to the external LD or TC cycles rather than being endogenous rhythms.

### Effect of environmental cycles on different body parts

**Fig. 4:**
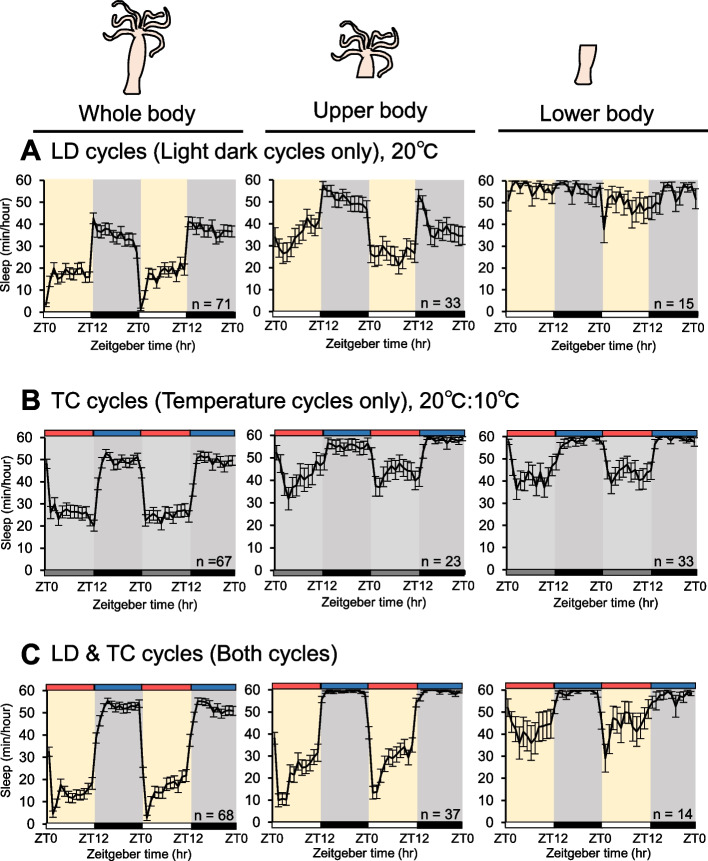
Differential response in the sleep-like state based on resection sites. Daily sleep profiles under (**A**) LD12:12 or (**B**) TC cycles (20 °C/10 °C) or (**C**) LD12:12 and TC cycles. Represent mean ± SEM (*n* = 14–71) Left (whole body), middle (upper body), and right (lower body) panels of *Hydra*

**Fig. 5:**
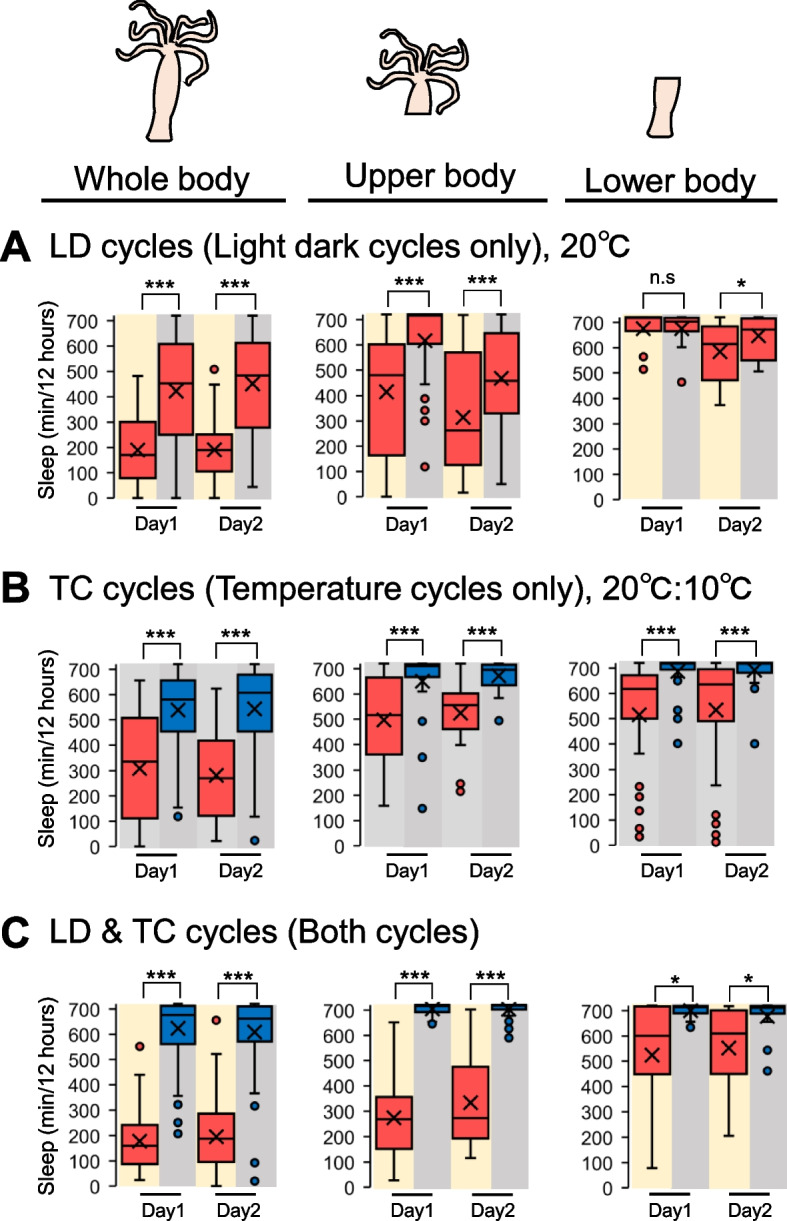
The total sleep under different environmental conditions. Box plots represent sleep amount (*n* = 14–71). **A** LD12:12 or **B** TC cycles (20 °C/10 °C) or **C** LD12:12 and TC cycles. Left (whole body), middle (upper body), and right (lower body) panels of *Hydra*. n.s., not significant. *P < 0.05, ***P < 0.001 by Wilcoxon signed-rank sum test [A–C]. LD12:12, 12-h/12-h light–dark; DD, constant darkness; TC, temperature cycles

Finally, we investigated which parts of the *Hydra* are responsible for receiving environmental cues to generate the sleep-like state using a resection method. The untreated, upper body, and lower body of *Hydra* were subjected to behavioral analyses under conditions of LD or TC cycles alone, as well as LDTC cycles were provided simultaneously. Before observing the effects of resection on the sleep-like state of *Hydra*, we first assessed whether the upper and lower body of a resected *Hydra* could move independently by quantifying their total activity levels. The results indicated that the activity of the resected *Hydra* was lower compared to the whole body. However, in our analysis, individuals with activity levels below a specific threshold were excluded from further analysis. Consequently, despite a reduction in activity, we determined that autonomous movement was still possible for both body segments (Supplementary Fig. 3). The results demonstrated that, under LD cycles conditions alone, upper body segments exhibited a sleep-like state comparable to that of the controls, with a statistically lower amount of sleep during the day and a higher amount of sleep during the night. However, no significant alterations in the amount of sleep were observed in the lower body of *Hydra* at Day1 (Figs. 4A and 5A). Surprisingly, under TC cycles conditions alone, all patterns exhibited a significantly lower amount of sleep during the warmer period and a higher amount of sleep during the cooler period (Figs. 4B and 5B). Conversely, when LD and TC cycles were provided simultaneously, the controls and upper body of *Hydra* exhibited a sleep-like state that were similar to those observed under a single environmental cue. Notwithstanding, notable discrepancies in the sleep-like state were observed between the initial day and night in the lower body of *Hydra* (Figs. 4C and 5C). These results indicate that the upper body of *Hydra* is able to alter the sleep-like state in response to environmental cycles under single environmental cue. Conversely, the lower body of *Hydra* is able to alter the sleep-like state under TC cycles, but not under a LD cycles.

**Fig. 6:**
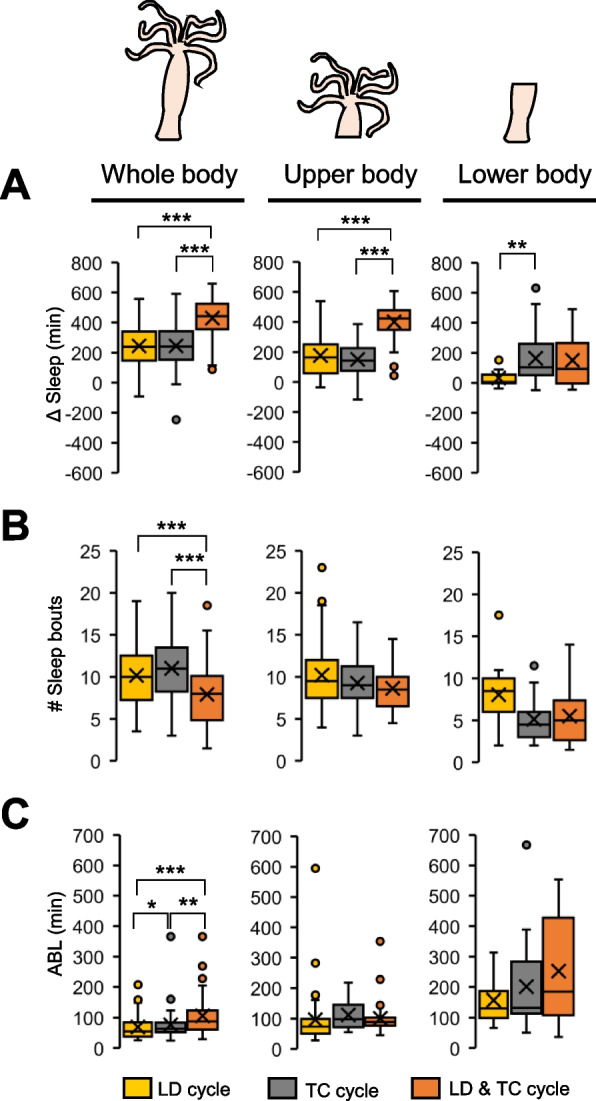
Simultaneous input of light and temperature enhance quality of the sleep-like state. Box plots represent **A** Δ Sleep (n = 14–71), **B** number of sleep bouts (*n* = 13–71), **C** ABL (*n* = 13–71). n.s., not significant. *P < 0.05, **P < 0.01, ***P < 0.001 by Kruskal–Wallis test followed by Mann–Whitney U test with Bonferroni correction [A-C]. LD12:12, 12-h/12-h light–dark; DD, constant darkness; TC, temperature cycles

We then examined Δ Sleep, which represents the change in the amount of sleep between the light and dark or 20 °C and 10 °C phases. The findings revealed that in all regions examined, Δ Sleep was significantly higher when exposed to LDTC cycles compared to other environmental conditions (Fig. 6A). We also examined the number of bouts and ABL. Consequently, a significant difference was observed in the number of bouts between LD, TC and LDTC cycles in intact *Hydra*, whereas no difference was evident in resected *Hydra* (Fig. 6B). Additionally, ABL differed among all environmental conditions in intact *Hydra*, with the longest ABL observed under LDTC cycles, followed by TC cycles conditions (Fig. 6C). Conversely, the upper and lower body of *Hydra* demonstrated no difference across conditions.

## Discussion

### The sleep-like state of *Hydra* under cold temperature

Ectotherms regulate their body temperature by selecting preferred temperature habitats through behavioral means [18, 19], as movement to suitable temperature zones serves as a means of regulating their own metabolic levels. The present study demonstrated that the total sleep of *Hydra* increased at 10 °C compared to 20 °C. This aligns with the general understanding that lower temperatures often result in reduced metabolic and physiological activity in ectotherms. For instance, *Hydra* enters a state of rest when exposed to temperatures around 4–6 °C, which is characterized by the dissolution of tentacles, the formation of a ball-like body, and a lack of responsiveness to stimuli [20]. Despite the cooler temperature, the *Hydra* did not enter a state of rest at 10 °C, as evidenced by their maintained sleep-like state in response to the LD cycles. This indicates that, while lower temperatures reduce activity and extend duration of the sleep-like state, the *Hydra* is not merely in a state of constant inactivity; rather, it continues to exhibit rhythmic patterns of the sleep-like state that are aligned with LD cycles. Additionally, the higher arousal threshold at 10 °C, as shown by a lower response to light stimuli, suggests that at cooler temperatures, *Hydra* may be in a deeper sleep-like state. These findings illustrate the intricate nature of sleep-like state regulation in *Hydra*, demonstrating that they are capable of regulating sleep-like state in response to environmental temperature, while maintaining sleep-like state in response to light cycles.

### The sleep-like state of *Hydra* under temperature cycles

It is well established that temperature acts as an environmental cue that influences behavior. For example, the fruit fly *Drosophila melanogaster* can respond to temperature cycles with a difference of 2 °C [10]. We have shown that *Hydra* were capable of changing its sleep-like state in response to temperature cycles, even in the absence of a traditional circadian clock. This response was evident with a 10 °C difference, but not with a 2 °C difference, indicating a threshold for temperature sensitivity. This threshold suggests that the *Hydra* use temperature changes as adaptive cues to regulate their sleep-like state, which reflects the natural environment’s significant temperature fluctuations. The interplay between the light and temperature cycles revealed that temperature cues might dominate when environmental signals are misaligned. *Hydra* exhibited a higher amount of sleep during the low-temperature phases, irrespective of light condition, highlighting the dominant influence of temperature on sleep-like state regulation. Furthermore, the observed increase in the number of sleep bouts, and shorter sleep latency under misaligned cycles indicate that mismatched environmental cues disrupt the efficiency of the sleep-like state in *Hydra*. Similar findings in *Drosophila* have shown that matching light and temperature conditions enhances rhythmic strength [21], suggesting that these organisms have evolved to adapt to natural environments where these cycles are aligned. These findings indicate that in *Hydra*, temperature is stronger environmental cue than light for regulating the sleep-like state. This preference for temperature cues is likely because *Hydra's* aquatic habitat is subject to significant temperature fluctuations, which can directly impact survival. As ectotherms, these organisms regulate their body temperature by selecting preferred temperature habitats through behavioral means [18, 19]. Additionally, a comparison of waking activity between 20 °C and 10 °C in 20 °C:10 °C TC cycles demonstrated that the waking activity of 10 °C was significantly lower than 20 °C (Supplementary Fig. 1). Therefore, the increase in the amount of sleep observed under TC cycles, especially during low temperature phases, may be attributed to a reduction in metabolic activity. This point requires careful consideration in future studies. Interestingly, studies in *Drosophila* have revealed that cold temperatures are sensed by a specialized neuronal circuit that regulate the duration of the sleep-like state, independently of metabolic changes [22]. These findings suggest that similar mechanisms may exist in *Hydra*, where not only do low temperatures reduce metabolic rates, but activity also influences the sleep-like state regulation through neural pathways. In future research, it will be necessary to develop methods that define the sleep-like state other than behavior to distinguish whether alteration in the sleep-like state due to temperature fluctuations are metabolism-dependent or not.

### Body parts responsible for receiving environmental cues

Previous studies have indicated the existence of specific behavioral controls by specific tissues/neurons, even in the diffuse nervous system of *Hydra* [17, 23, 24]. This suggests the possible existence of a central mechanism for the sleep-like states regulation, even in the absence of a central nervous system. Our experiments on resected *Hydra* with LD and TC cycles revealed notable insights into their region-specific responses to these environmental cues. Different body parts of *Hydra* appear to have varying sensitivities to LD and TC cycles. In particular, upper body of *Hydra* demonstrated the capacity to transition into the sleep-like state with LD cycles, whereas lower body of *Hydra* cannot. Furthermore, intact *Hydra* and upper body of *Hydra*, ΔSleep was significantly higher when exposed to LDTC cycles than when exposed to a single environmental cue. However, in the lower body of *Hydra*, there was no difference in ΔSleep between the TC and the LDTC cycles. This suggests that the light reception in *Hydra* is likely to be localized to the head. Previous reports have indicated the high expression of two opsins, *HvOpC5* and *HvOpD1*, out of 45 types in the vicinity of the head, along with the presence of phototransduction-related proteins. Therefore, the head is considered to be the photoreceptor organ that establishes the sleep-like state mediated by LD cycles [13]. Additionally, previous study reported that PRKG1, melatonin, GABA, and dopamine acting as regulator of the sleep-like state under LD cycles in *Hydra*. Expression of PRKG1 has been identified at the mRNA level in the head region [5], and dopamine has been shown to be expressed in the hypostome of the head via antibody staining [25]. These findings suggest that PRKG1 and dopamine may play roles in regulating the sleep-like state of *Hydra* in response to light. Conversely, under the TC cycles, both the upper and lower body of *Hydra* exhibited a sleep-like state in response to environmental cycles. Based on these results, its suggest that the temperature sensitivity for regulating the sleep-like state of *Hydra* is systemic. one recent study reported that temperature-coupled contraction burst (tCB) neurons located in the peduncle of *Hydra* are involved in temperature-stimulus-dependent behavior [15]. In addition, *Hydra* has been reported to possess 34 types of transient receptor potential (TRP) channels [14]. Future research should aim to identify which temperature receptors or temperature-sensing cells are necessary for regulating the sleep-like state in *Hydra*. Additionally, it is important to investigate the role of known regulators of the sleep-like state of *Hydra* in LDTC cycles.

For instance, contraction behavior involves two networks in the head and foot regions. Disruption of each network resulted in a reduction in responsiveness to stimuli [17]. Therefore, resected *Hydra* may have behavioral limitations. Further study is needed to categorize the behavior of resected *Hydra* as indicated in a recent report [26]. However, lower body of *Hydra* was capable of changing their sleep-like state to some extent when exposed to LDTC or TC cycles. This suggests that, while the foot side may be important for contraction behavior, it can still exhibit autonomous behavior/sleep patterns under sufficient environmental cues.

## Conclusions

In summary, this study highlights the multifaceted role of temperature in the regulation of the sleep-like state in *Hydra*. Our findings suggest that temperature not only influences sleep duration, but also plays a critical role in the regulation of the sleep-like state in response to environmental cycles. Moreover, the differential responses of resected *Hydra* to light and temperature cues provide insights into the potential neural and sensory mechanisms underlying the regulation of the sleep-like state in this simple organism. These findings pave the way for further research on the molecular and physiological basis of sleep-like states in cnidarians and other simple animals, offering a broader understanding of sleep evolution and regulation across the animal kingdom.

## Supplementary Information

Supplementary Material 1: Supplemental Fig 1. The waking activity of *Hydra* under TC cycles (20 °C/10 °C). A) Daily average waking activity profiles under TC cycles (20 °C/10 °C). Represent mean ± SEM (*n* = 99) B) Mean waking activity over two experimental days in 20 °C and 10 °C.***P < 0.001, by Wilcoxon signed rank test.

Supplementary Material 2: Supplemental Fig 2. The sleep-like state under constant environment. A) Daily sleep profiles under 20 °C /10 °C temperature cycles and LD cycles for three days followed by 2 days of constant temperature (10 °C) and constant darkness. Represent mean ± SEM (*n* = 26). B) Boxplots represent sleep amount in each 12 h bin. C) Daily sleep profiles under 20 °C /10 °C temperature cycles and LD cycles for two days followed by three days of constant temperature (20 °C) and constant light. Represent mean ± SEM (*n* = 14). D) Boxplots represent sleep amount in each 12 h bin. n.s., not significant., **P < 0.01, and ***P < 0.001, by Wilcoxon signed rank test.

Supplementary Material 3: Supplemental Fig. 3. The total activity of resected *Hydra* in various conditions. Daily total activity profiles of whole body (Gray line), upper body (Purple line), and lower body (Green line) under (A) LD12:12 or (B) TC cycles (20 °C/10 °C) or (C) LD12:12 and TC cycles. Represent mean ± SEM (*n* = 13–71). Mean total activity level (E) LD12:12 or (F) TC cycles (20 °C/10 °C) or (G) LD12:12 and TC cycles. n.s., not significant., ***P < 0.001, by Kruskal–Wallis test followed by Dunn test (Bonferroni correction).

## Data Availability

The datasets during and/or analyzed during the current study are available from the corresponding author on reasonable request.

## Abbreviations

HCS: Hydra culture solution
LD: Light–dark
ABL: Average sleep bout length
TC: Temperature cycles
LDTC: Both LD and TC cycles
tCB: Temperature-coupled contraction burst
TRP: Transient receptor potential
