# Supplementary material for: Effect of temperature cycles on the sleep-like state in Hydra vulgaris
Source: Zoological Lett. 2025 Jan 28;11:2. doi: 10.1186/s40851-025-00248-1 (PMC11773864; doi:10.1186/s40851-025-00248-1)
Supplement: Supplementary file 1 — Supplementary Material 1: Supplemental Fig 1. The waking activity of Hydra under TC cycles (20 °C/10 °C). A) Daily average waking activity profiles under TC cycles (20 °C/10 °C). Represent mean ± SEM (n = 99) B) Mean waking activity over two experimental days in 20 °C and 10 °C.***P < 0.001, by Wilcoxon signed rank test. [file 40851_2025_248_MOESM1_ESM.pdf]

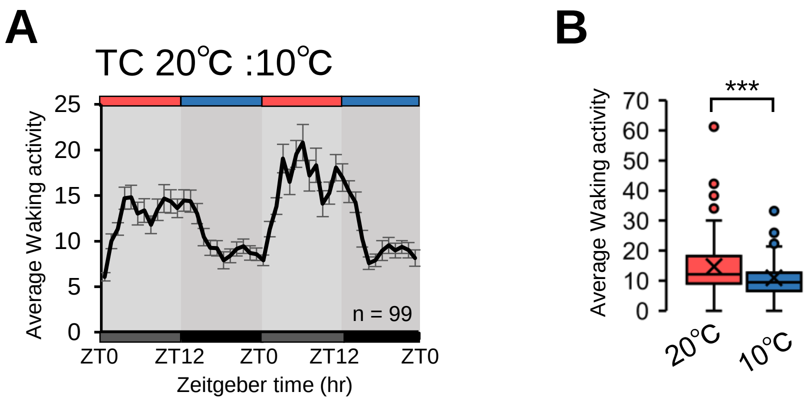

Supplemental figure 1 The waking activity of *Hydra* under TC cycles (20° C/10° C)

A) Daily average waking activity profiles under TC cycles (20° C/10° C). Represent mean ± SEM (n = 99)

B) Mean waking activity over two experimental days in 20° C and 10° C. $P < 0.001$, by Wilcoxon signed rank test.
