# Supplementary material for: Effect of temperature cycles on the sleep-like state in Hydra vulgaris
Source: Zoological Lett. 2025 Jan 28;11:2. doi: 10.1186/s40851-025-00248-1 (PMC11773864; doi:10.1186/s40851-025-00248-1)
Supplement: Supplementary file 2 — Supplementary Material 2: Supplemental Fig 2. The sleep-like state under constant environment. A) Daily sleep profiles under 20 °C /10 °C temperature cycles and LD cycles for three days followed by 2 days of constant temperature (10 °C) and constant darkness. Represent mean ± SEM (n = 26). B) Boxplots represent sleep amount in each 12 h bin. C) Daily sleep profiles under 20 °C /10 °C temperature cycles and LD cycles for two days followed by three days of constant temperature (20 °C) and constant light. Represent mean ± SEM (n = 14). D) Boxplots represent sleep amount in each 12 h bin. n.s., not significant., **P < 0.01, and ***P < 0.001, by Wilcoxon signed rank test. [file 40851_2025_248_MOESM2_ESM.pdf]

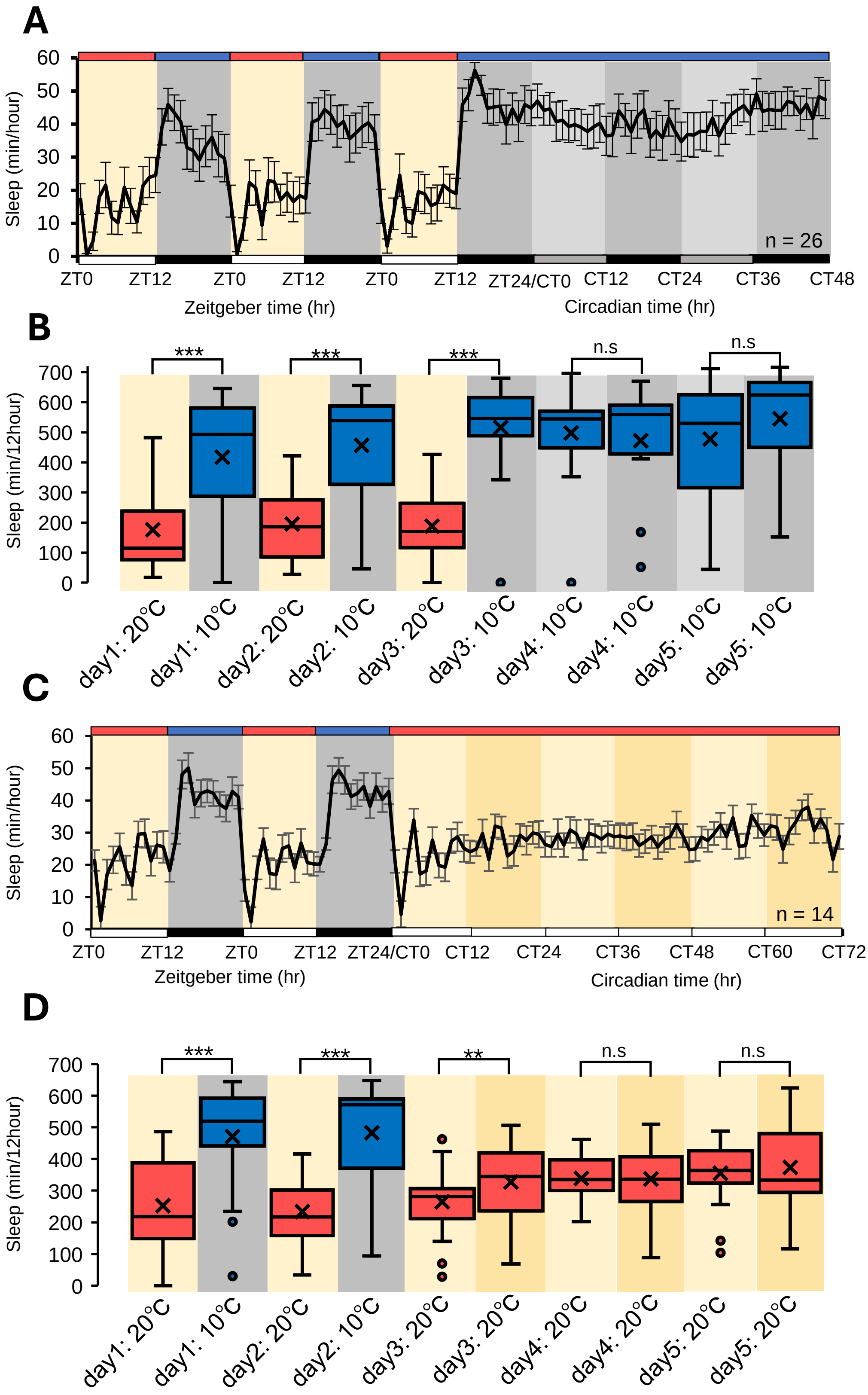

Supplemental figure 2 The sleep-like state under constant environment
A) Daily sleep profiles under 20 ° C /10° C temperature cycles and LD cycles for 3 days followed by 2 days of constant temperature (10° C) and constant darkness. Represent mean ± SEM (n = 26). B) Boxplots represent sleep amount in each 12hr bin. C) Daily sleep profiles under 20 ° C /10° C temperature cycles and LD cycles for 2 days followed by 3 days of constant temperature (20° C) and constant light. Represent mean ± SEM (n = 14). D) Boxplots represent sleep amount in each 12hr bin. n.s., not significant., **P < 0.01, and ***P < 0.001, by Wilcoxon signed rank test.
