# Supplementary material for: Effect of temperature cycles on the sleep-like state in Hydra vulgaris
Source: Zoological Lett. 2025 Jan 28;11:2. doi: 10.1186/s40851-025-00248-1 (PMC11773864; doi:10.1186/s40851-025-00248-1)
Supplement: Supplementary file 3 — Supplementary Material 3: Supplemental Fig. 3. The total activity of resected Hydra in various conditions. Daily total activity profiles of whole body (Gray line), upper body (Purple line), and lower body (Green line) under (A) LD12:12 or (B) TC cycles (20 °C/10 °C) or (C) LD12:12 and TC cycles. Represent mean ± SEM (n = 13–71). Mean total activity level (E) LD12:12 or (F) TC cycles (20 °C/10 °C) or (G) LD12:12 and TC cycles. n.s., not significant., ***P < 0.001, by Kruskal–Wallis test followed by Dunn test (Bonferroni correction). [file 40851_2025_248_MOESM3_ESM.pdf]

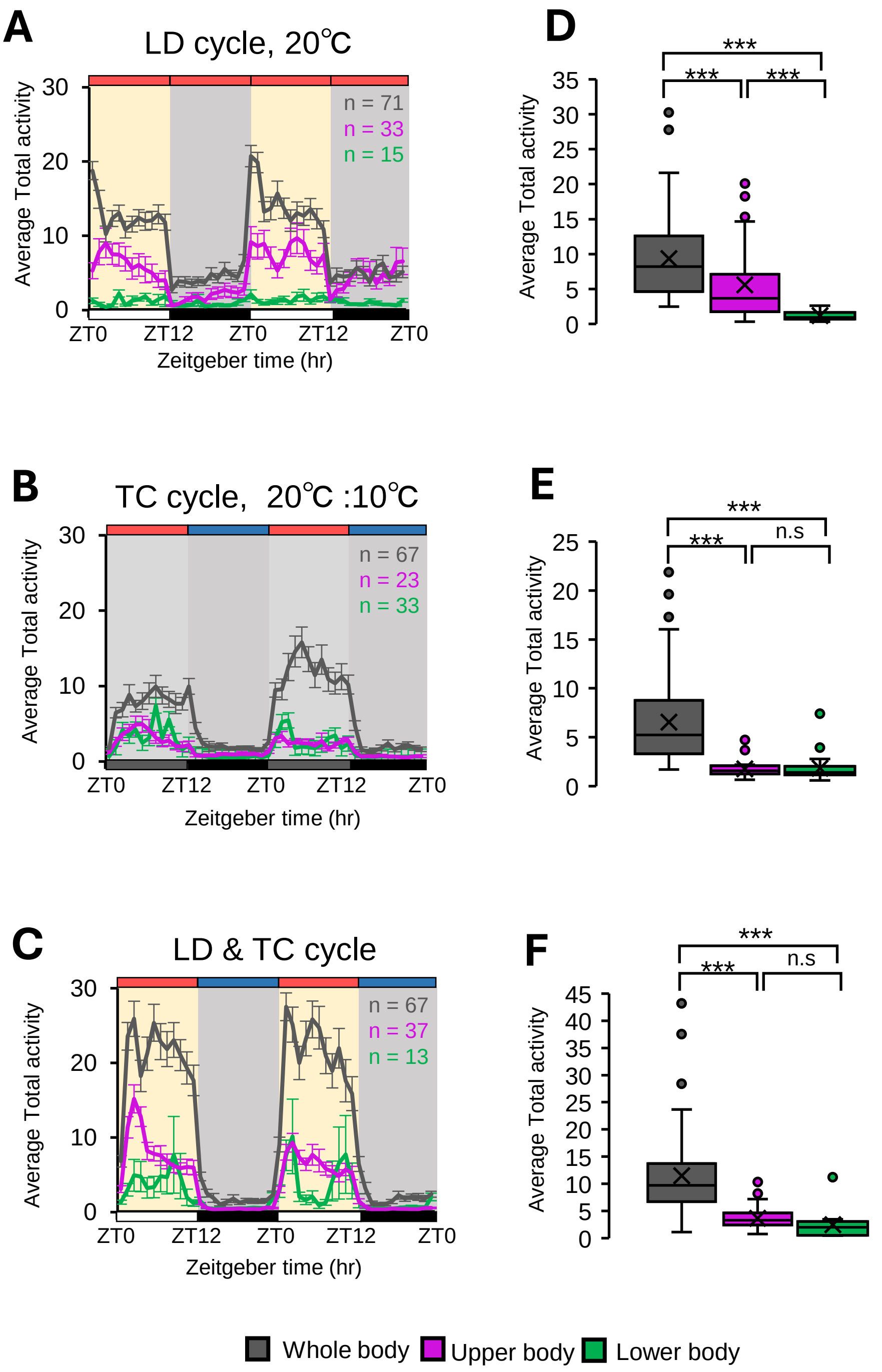

Supplemental figure 3 The total activity of resected *Hydra* in various conditions
Daily total activity profiles of whole body (Gray line), upper body (Purple line), lower body (Green line) under (A) LD12:12 or (B) TC cycles (20° C/10° C) or (C) LD12:12 and TC cycles. Represent mean ± SEM (n = 13-71). Mean total activity over two experimental days (E) LD12:12 or (F) TC cycles (20° C/10° C) or (G) LD12:12 and TC cycles. n.s., not significant., ***P < 0.001, by Kruskal-Wallis test followed by Dunn test (Bonferroni correction).
